# Supplementary material for: Clinical Cholera Surveillance Sensitivity in Bangladesh and Implications for Large-Scale Disease Control
Source: J Infect Dis. 2021 Aug 28;224(Suppl 7):S725–31. doi: 10.1093/infdis/jiab418 (PMC8687068; doi:10.1093/infdis/jiab418)

**Supplementary Figure 1.** A. Median risk of *V. cholerae* seroincidence relative to a population-weighted mean by 5 km x 5 km grid cell. These relative risk estimates are bounded such that RRs above and below 2 and -2 were plotted as the values 2 and -2, respectively. The black marks indicate sentinel hospital locations. B. Median number of estimated V. cholerae infections per grid cell in the previous year. The black marks indicate sentinel hospital locations. These estimates were calculated as the product of the median seroincidence risk and 2015 WorldPop population estimate in each grid cell. The black marks indicate sentinel hospital locations.


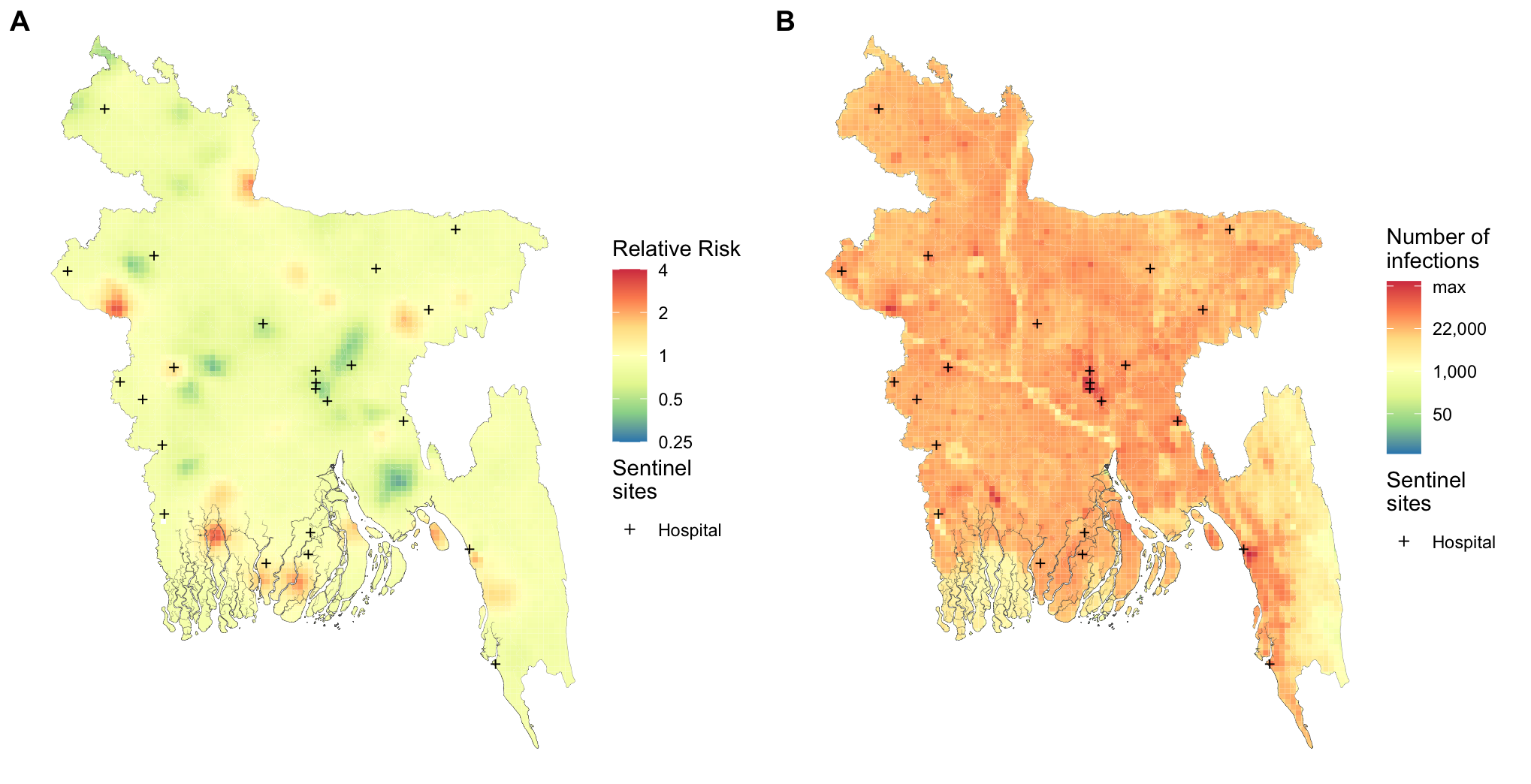

Supplement: jiab418_suppl_Supplementary_Figure_S1 [file jiab418_suppl_supplementary_figure_s1.docx]
