# Supplementary material for: Clinical Cholera Surveillance Sensitivity in Bangladesh and Implications for Large-Scale Disease Control
Source: J Infect Dis. 2021 Aug 28;224(Suppl 7):S725–31. doi: 10.1093/infdis/jiab418 (PMC8687068; doi:10.1093/infdis/jiab418)
Supplement: jiab418_suppl_Supplementary_Figure_S2 [file jiab418_suppl_supplementary_figure_s2.docx]

**Supplementary Figure 2.** A map of the cholera greyspots in Bangladesh if all 491 possible healthcare facilities from the sentinel site allocation strategy are used to establish the cholera surveillance zone. Populations living in the coral pink areas are inside the cholera surveillance zone. The grey areas are places where we would have little information on clinical cases of cholera in Bangladesh.

**
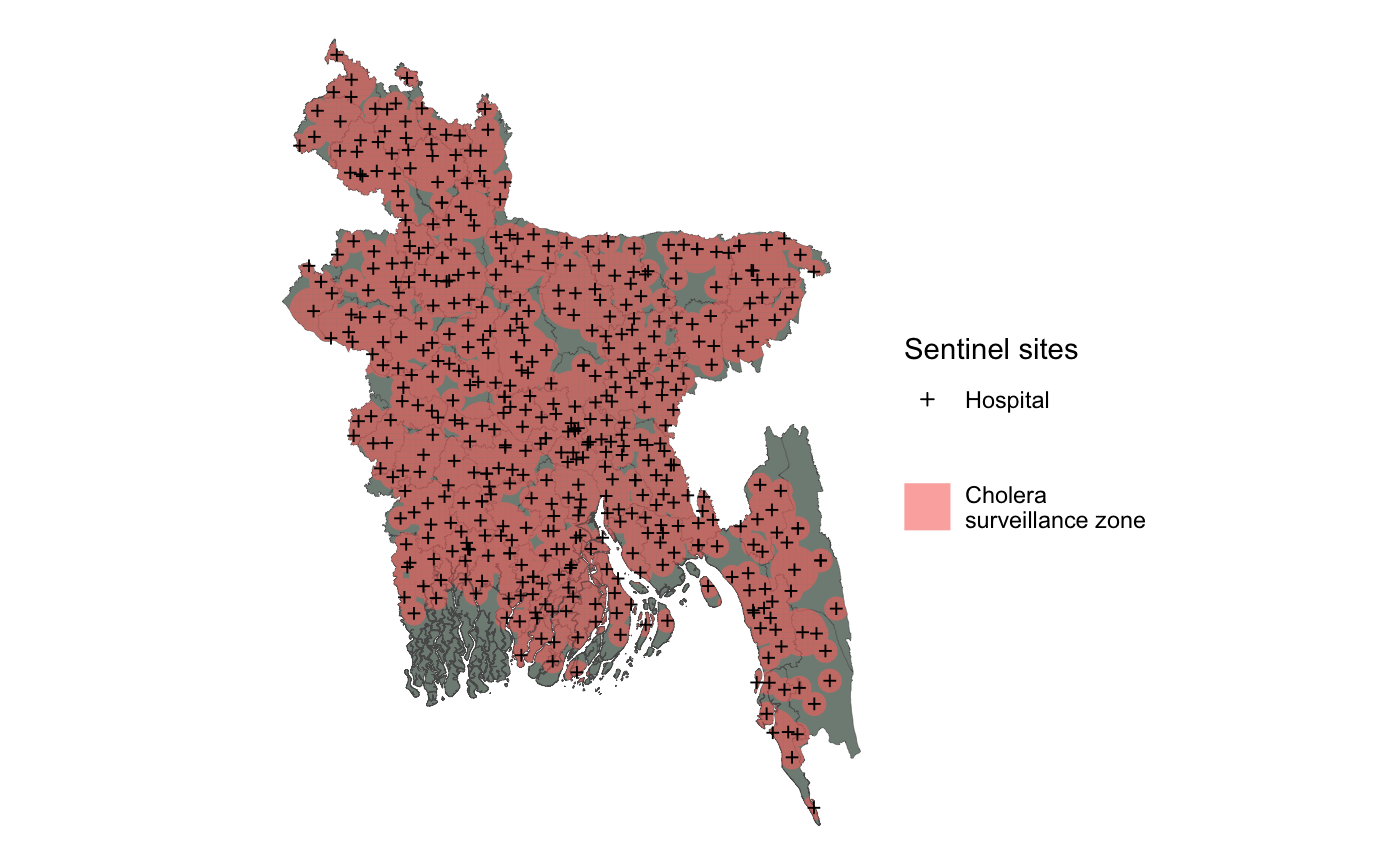
**
