# Supplementary material for: Clinical Cholera Surveillance Sensitivity in Bangladesh and Implications for Large-Scale Disease Control
Source: J Infect Dis. 2021 Aug 28;224(Suppl 7):S725–31. doi: 10.1093/infdis/jiab418 (PMC8687068; doi:10.1093/infdis/jiab418)
Supplement: jiab418_suppl_Supplementary_Materials [file jiab418_suppl_supplementary_materials.docx]

### **Supplementary Methods**

*Data*

To assess the sensitivity of the cholera surveillance system, we used previously published, modeled *V. cholerae* estimates of seroincidence based on a nationally-representative serosurvey conducted in 70 communities in Bangladesh in 2015 [[1]](https://paperpile.com/c/4CWYkH/99MW). Serum samples from 2,778 participants were tested using eight *V. cholerae*-specific assays to quantify vibriocidal titers (Ogawa and Inaba), anti-LPS IgG and IgA antibodies (maximum of Inaba and Ogawa for each person) and anti-CTB IgG and IgA antibodies. These data were then used in a previously validated machine learning model [[2]](https://paperpile.com/c/4CWYkH/nJEgG) to estimate the annual seroincidence rate. To generate high-resolution national maps of infection risk, a Bayesian geostatistical model adjusting for population characteristics like age and sex was used. This estimated the risk of infection in the year prior to data collection relative to the population-weighted mean across a 5 km x 5km grid of Bangladesh. More information on the model specifics can be found at Azman and Lauer et al [[1]](https://paperpile.com/c/4CWYkH/99MW).

*Examining alternative sentinel site selection with a simulation-based approach*

We filtered public hospitals in the Bangladesh Ministry of Health and Family Welfare Facility Registry of the following types: 200-250 bed, 300-500 bed, general, district, medical college, and upazila health complex [[3]](https://paperpile.com/c/4CWYkH/FF0ZB).  We then used the R package tidygeocoder to obtain geographic coordinates for each hospital using the Google Earth and the Open Street Maps APIs [[4]](https://paperpile.com/c/4CWYkH/uSVgL). For each facility, we performed multiple English language searches with the facility name, “Bangladesh,” and different combinations of the division, district, and upazila names. Out of 504 possible hospitals, 491 were successfully geocoded.

One control strategy selected sites randomly from all facilities (Random), while another selected sites to match the number of sentinel sites by first-level administrative units (Division). Beyond the “Random” and “Division” strategies, site selection differed critically in two dimensions: 1) the key indicator used to rank sentinel sites (i.e., population density, mean relative risk of seroincidence in the presumed catchment area, or total absolute seroincidence risk in the presumed catchment area); and 2) the guiding principle behind the spatial distribution of sentinel sites (i.e., “Division” - match the distribution across divisions, “Equity”- at least one site per division before optimizing by site along the key indicator). Twenty-two new sites were selected for each set.

The strategies we compared are as follows:

- Random selection: Select sites randomly. This is a negative control.
- Division selection: Select sites randomly within divisions while matching the current distribution of facilities by division (largest sub-national administrative unit). This is a geographically-stratified negative control.
- Population-Division selection: Within divisions, select sites weighted by population density in their prospective cholera surveillance zones (i.e., Calculate the population density in the 10, 20, and 30 km buffer around upazila, district, and tertiary care facilities, respectively). Match the current distribution of facilities by division.
- Population-Equity selection: There must be at least one site in each of the eight divisions in Bangladesh and these are weighted by population density in the site’s prospective cholera surveillance zone. All remaining sites are selected by population density weight without constraints on division.
- Relative Risk-Division selection: Within divisions, select sites weighted by mean relative risk of seroincidence in their prospective cholera surveillance zones (i.e. Calculate the mean relative risk across all cells in the buffer zone). Match the current distribution of facilities by division.
- Relative Risk-Equity selection: There must be at least one site in each of the eight divisions in Bangladesh and these are weighted by mean relative risk of seroincidence in the site’s prospective cholera surveillance zone. All remaining sites are selected by relative risk weight without constraints on division.
- Absolute Risk-Division selection: Within divisions, select sites weighted by total absolute risk of seroincidence in their prospective cholera surveillance zones (i.e. Calculate the sum of median estimated infections across all cells in the buffer zone). Match the current distribution of facilities by division.
- Absolute Risk-Equity selection: There must be at least one site in each of the eight divisions in Bangladesh and these are weighted by total absolute risk of seroincidence in the site’s prospective cholera surveillance zone. All remaining sites are selected by total absolute risk weight without constraints on division.

*References*

1. [Azman AS, Lauer SA, Bhuiyan TR, et al. Vibrio cholerae O1 transmission in Bangladesh: insights from a nationally representative serosurvey [Internet]. The Lancet Microbe. 2020. p. e336–e343. Available from:](http://paperpile.com/b/4CWYkH/99MW) <http://dx.doi.org/10.1016/s2666-5247(20)30141-5>

2. [Azman AS, Lessler J, Luquero FJ, et al. Estimating cholera incidence with cross-sectional serology. Sci Transl Med [Internet]. **2019**; 11(480). Available from:](http://paperpile.com/b/4CWYkH/nJEgG) <http://dx.doi.org/10.1126/scitranslmed.aau6242>

3. [Government of Bangladesh. Facility Registry [Internet]. [cited 2021 May 13]. Available from:](http://paperpile.com/b/4CWYkH/FF0ZB) <http://facilityregistry.dghs.gov.bd/index.php>

4. [Cambon J. tidygeocoder [Internet]. Github; [cited 2021 May 13]. Available from:](http://paperpile.com/b/4CWYkH/uSVgL) <https://github.com/jessecambon/tidygeocoder>
