# Supplementary material for: Clinical Cholera Surveillance Sensitivity in Bangladesh and Implications for Large-Scale Disease Control
Source: J Infect Dis. 2021 Aug 28;224(Suppl 7):S725–31. doi: 10.1093/infdis/jiab418 (PMC8687068; doi:10.1093/infdis/jiab418)
Supplement: jiab418_suppl_Supplementary_Table_S1 [file jiab418_suppl_supplementary_table_s1.docx]

**Supplementary Table 1.** Healthcare facilities in Bangladesh that perform laboratory confirmation of *V. cholerae.*

| **Hospital** | **Division** | **Type** |
| --- | --- | --- |
| Upazila Health Complex Bakerganj | Barisal | subdistrict |
| Upazila Health Complex Mathbariya | Barisal | subdistrict |
| General Hospital Patuakhali | Barisal | tertiary |
| District Sadar Hospital Cox's Bazar | Chittagong | district |
| General Hospital Comilla | Chittagong | district |
| Bangladesh Institute of Tropical and Infectious Diseases Chittagong | Chittagong | tertiary |
| District Hospital Norshingdi | Dhaka | district |
| General Hospital Narayanganj | Dhaka | district |
| General Hospital Tangail | Dhaka | district |
| Dhaka Medical College Dhaka | Dhaka | tertiary |
| Uttara Adhunik Medical College Hospital | Dhaka | tertiary |
| District Sadar Hospital Satkhira | Khulna | district |
| General Hospital Kusthia | Khulna | district |
| General Hospital Meherpur | Khulna | district |
| Sadar Hospital Chuadanga | Khulna | district |
| Upazila Health Complex Chaugachha Jesssore | Khulna | subdistrict |
| Upazila Health Complex Madan | Mymensingh | subdistrict |
| Adhunik Sadar Hospital Naogaon | Rajshahi | district |
| Health Complex Shibganj | Rajshahi | subdistrict |
| Adhunik Sadar Hospital Thakurgaon | Rangpur | district |
| Adhunik Sadar Hospital Habiganj | Sylhet | district |
| Upazila Health Complex Chhatak Sunamganj | Sylhet | subdistrict |
