# Supplementary material for: Clinical Cholera Surveillance Sensitivity in Bangladesh and Implications for Large-Scale Disease Control
Source: J Infect Dis. 2021 Aug 28;224(Suppl 7):S725–31. doi: 10.1093/infdis/jiab418 (PMC8687068; doi:10.1093/infdis/jiab418)
Supplement: jiab418_suppl_Supplementary_Table_S2 [file jiab418_suppl_supplementary_table_s2.docx]

**Supplementary Table 2.** A. The total number and percent of infections that may be captured in the cholera surveillance zone. The percent infected represents the percentage of infected individuals captured within the cholera surveillance zone out of all infected individuals in Bangladesh. B. The number and percent of infections that may be captured in the cholera surveillance zone categorized by the different risk metrics used, relative risk and absolute risk. The infections in the surveillance zone represent the percentage of infected people in high-moderate-low risk grid cells among all infections within the cholera surveillance zone. The population in the surveillance zone represents the percentage of people living in high-moderate-low risk grid cells among all people within the cholera surveillance zone. C. The number and percent of infections that may be captured across Bangladesh categorized by the different risk metrics used, relative risk and absolute risk. The captured at-risk population represents the percentage of high-moderate-low risk populations captured by the cholera surveillance zone out of all high-moderate-low risk populations in Bangladesh. The captured infections represent the percentage of infections in high-moderate-low risk grid cells among all infections in high-moderate-low risk grid cells across Bangladesh.

**A.** *Total cholera surveillance zone population and infections*

| Geographic frame | Population (in millions) | Number infected (in millions) | Percent infected (%) | Percent of all infections in Bangladesh (%) |
| --- | --- | --- | --- | --- |
| Cholera surveillance zone | 50.9 | 8.0 (4.6, 11.9) | 15.7 (13.3 , 23.4) | 28.5 (26.6 , 34.6) |

**B.** *Population and infections captured in the cholera surveillance zone by risk category*

| Risk category | Number infected (in millions) | Surveillance zone infections (%) | Surveillance zone population (in millions) | Population in surveillance zone (%) |
| --- | --- | --- | --- | --- |
| ***Relative risk*** | | | | |
| High | 3.4 (1.8, 6.0) | 43.4 (36.5 , 65) | 10.1 (5.6, 16.5) | 19.8 (16, 32.3) |
| Moderate | 3.6 (1.6, 6.0) | 44.4 (39.4, 58.5) | 23.9 (17.0, 31.5) | 47.0 (41.3, 61.8) |
| Low | 0.99 (0.22, 2.4) | 12.2 (8.0, 26.4) | 16.9 (8.5, 26.3) | 33.3 (26.4, 51.7) |
| ***Absolute risk*** | | | | |
| High | 6.2 (3.5, 9.2) | 77.0 (74.2, 85.9) | 32.8 (27.5, 36.8) | 64.5 (61.6, 72.3) |
| Moderate | 1.8 (0.76, 2.8) | 22.3 (19.6, 30.6) | 16.5 (13.3, 20.4) | 32.5 (29.8, 40.2) |
| Low | 0.062 (0.021, 0.11) | .77 (.6, 1.3) | 1.5 (0.54, 3.6) | 3.0 (1.9, 7.1) |

**C.** *Population and infections captured in Bangladesh by risk category*

| Risk category | Captured at-risk population (%) | Captured infections (%) |
| --- | --- | --- |
| ***Relative risk*** | | |
| High | 26.3 (22.4, 38.1) | 25.9 (21.6, 39.0) |
| Moderate | 30.1 (27.5, 36.7) | 29.7 (27, 36.8) |
| Low | 36.5 (31.9, 49.0) | 35.7 (31.6, 47) |
| ***Absolute risk*** | | |
| High | 41.9 (40.6, 45.8) | 33.3 (30.1, 42.3) |
| Moderate | 22.0 (20.5, 26.5) | 19.8 (19, 22.1) |
| Low | 15.8 (11.5, 27.8) | 10.8 (9.1, 15.5) |
