# Supplementary material for: Clinical Cholera Surveillance Sensitivity in Bangladesh and Implications for Large-Scale Disease Control
Source: J Infect Dis. 2021 Aug 28;224(Suppl 7):S725–31. doi: 10.1093/infdis/jiab418 (PMC8687068; doi:10.1093/infdis/jiab418)
Supplement: jiab418_suppl_Supplementary_Table_S3 [file jiab418_suppl_supplementary_table_s3.docx]

**Supplementary Table 3.** Mean and 95% CI for the number and percentage of the population and infections captured in the cholera surveillance zone across all sentinel site selection strategies.

| Strategy | Millions living in the surveillance zone (95% CI) | Percentage living in the surveillance zone (95% CI) | Millions of infections in the surveillance zone  (95% CI) | Percentage of infections in the surveillance zone (95% CI) |
| --- | --- | --- | --- | --- |
| Random | 53.1 (46.0, 59.8) | 32.7 (28.3, 36.8) | 8.3 (4.5, 12.5) | 15.6 (8.6, 23.1) |
| Division | 56.5 (52.2, 61.9) | 34.7 (32.1, 38.1) | 9.1 (5.0, 13.5) | 16.1 (9.0, 23.6) |
| Population Division | 52.4 (47.7, 58.6) | 32.2 (29.4, 36.1) | 8.5 (4.7, 12.6) | 16.2 (9.1, 23.7) |
| Population Equity | 53.3 (48.3, 61.3) | 32.8 (29.7, 37.7) | 8.2 (4.4, 12.4) | 15.3 (8.4, 22.6) |
| Relative Risk Division | 53.3 (50.0, 55.9) | 32.8 (30.8, 34.4) | 9.3 (5.3, 13.4) | 17.4 (10.1, 25.1) |
| Relative Risk Equity | 50.3 (48.2, 52.0) | 30.9 (29.6, 32.0) | 8.9 (5.2, 12.9) | 17.8 (10.4, 25.5) |
| Absolute Risk Division | 52.4 (49.1, 55.3) | 32.2 (30.2, 34.0) | 8.9 (5.1, 12.9) | 17.0 (9.9, 24.5) |
| Absolute Risk Equity | 55.4 (49.8, 60.9) | 34.1 (30.6, 37.5) | 9.2 (5.3, 13.4) | 16.6 (9.5, 24.0) |
