# Supplementary material for: Clinical Cholera Surveillance Sensitivity in Bangladesh and Implications for Large-Scale Disease Control
Source: J Infect Dis. 2021 Aug 28;224(Suppl 7):S725–31. doi: 10.1093/infdis/jiab418 (PMC8687068; doi:10.1093/infdis/jiab418)
Supplement: jiab418_suppl_Supplementary_Table_S4 [file jiab418_suppl_supplementary_table_s4.docx]

**Supplementary Table 4.** Intracluster correlation coefficients (ICC) for the number of infections in the cholera surveillance zone regressed against different sets of random effects. The purpose of these different models was to partition variability from the underlying seroincidence estimates (1000 posterior draws) and variability from the simulations of each strategy (20 simulations). Three random effects (RE) models were examined: 1) a random effect for each seroincidence posterior draw, 2) a random effect for each simulation in a given strategy, and 3) random effects for both seroincidence posterior draw and simulation.

| Strategy | Seroincidence RE | Simulation RE | Seroincidence and Simulation REs |
| --- | --- | --- | --- |
| Random | 0.84 | 0.10 | 0.94 |
| Division | 0.89 | 0.06 | 0.95 |
| Population Division | 0.92 | 0.05 | 0.97 |
| Population Equity | 0.87 | 0.10 | 0.97 |
| Relative Risk Division | 0.96 | 0.02 | 0.98 |
| Relative Risk Equity | 0.97 | 0.01 | 0.98 |
| Absolute Risk Division | 0.95 | 0.02 | 0.97 |
| Absolute Risk Equity | 0.94 | 0.04 | 0.98 |
